# A Review of the Current Impact of Inhibitors of Apoptosis Proteins and Their Repression in Cancer

**DOI:** 10.3390/cancers14071671

**Published:** 2022-03-25

**Authors:** Pierina Cetraro, Julio Plaza-Diaz, Alex MacKenzie, Francisco Abadía-Molina

**Affiliations:** 1 Research and Advances in Molecular and Cellular Immunology, Center of Biomedical Research, University of Granada, Armilla, 18016 Granada, Spain; piericetraro@gmail.com; 2 Department of Biochemistry and Molecular Biology II, School of Pharmacy, University of Granada, 18071 Granada, Spain; 3 Children’s Hospital of Eastern Ontario Research Institute, Ottawa, ON K1H 8L1, Canada; mackenzie@cheo.on.ca; 4 Instituto de Investigación Biosanitaria IBS.GRANADA, Complejo Hospitalario Universitario de Granada, 18014 Granada, Spain; 5 Department of Cellular and Molecular Medicine, Faculty of Medicine, University of Ottawa, Ottawa, ON K1H 8M5, Canada; 6 Institute of Nutrition and Food Technology “José Mataix”, Biomedical Research Center, University of Granada, Armilla, 18016 Granada, Spain; 7 Department of Cell Biology, School of Sciences, University of Granada, 18071 Granada, Spain

**Keywords:** inhibitor of apoptosis proteins, SMAC mimetics, apoptosis, NF-κB, TNF-α, endoplasmic reticulum stress

## Abstract

**Simple Summary:**

The Inhibitor of Apoptosis (IAP) family of proteins has emerged as a potential pharmacological target in cancer. Abnormal expression of IAPs can lead to dysregulated cell suicide, promoting the development of different pathologies. In several cancer types, members of this protein family are overexpressed while their natural antagonist (Smac) appears to be downregulated, contributing to the acquisition of resistance to traditional therapy. The development of compounds that mimic the action of Smac showed promise in the re-sensitization of the cell suicide defense mechanism in cancer cells, particularly in combination with other treatments. Interaction with other molecules, such as tumor necrosis factor-α, in the tumor microenvironment reveals a complex interplay between IAPs and cancer.

**Abstract:**

The Inhibitor of Apoptosis (IAP) family possesses the ability to inhibit programmed cell death through different mechanisms; additionally, some of its members have emerged as important regulators of the immune response. Both direct and indirect activity on caspases or the modulation of survival pathways, such as nuclear factor kappa-light-chain-enhancer of activated B cells (NF-κB), have been implicated in mediating its effects. As a result, abnormal expression of inhibitor apoptosis proteins (IAPs) can lead to dysregulated apoptosis promoting the development of different pathologies. In several cancer types IAPs are overexpressed, while their natural antagonist, the second mitochondrial-derived activator of caspases (Smac), appears to be downregulated, potentially contributing to the acquisition of resistance to traditional therapy. Recently developed Smac mimetics counteract IAP activity and show promise in the re-sensitization to apoptosis in cancer cells. Given the modest impact of Smac mimetics when used as a monotherapy, pairing of these compounds with other treatment modalities is increasingly being explored. Modulation of molecules such as tumor necrosis factor-α (TNF-α) present in the tumor microenvironment have been suggested to contribute to putative therapeutic efficacy of IAP inhibition, although published results do not show this consistently underlining the complex interaction between IAPs and cancer.

## 1. Introduction

Many cancers demonstrate evasion of apoptosis enhancing cell survival [1]. Alteration in structure, expression, or even epigenetic regulation of genes, such as *TP53* or *BCL2* and the caspase-family of genes (or their products) leading to a dysregulated apoptotic process, are classically involved in this hallmark of cancer [2]. Dysregulated apoptosis is often observed in other pathological conditions, such as neurodegenerative disorders (Alzheimer’s Disease, motor system disorders, stroke and many others) or autoimmune diseases (type I diabetes, multiple sclerosis, rheumatoid arthritis and more) [3,4]. Many molecular pathways participating in this dysregulation have been associated with resistance to anticancer therapy that are based on the induction of endogenous cell death mechanisms [5]. Therefore, new therapeutic approaches aiming at the re-engagement of the apoptotic process by resistant cells are promising strategies to combat cancer.

The Inhibitor of Apoptosis Protein (IAP) family is a group of apoptosis negative regulators, characterized by the presence of at least one copy of the Baculovirus IAP Repeat (BIR) domain in their N-terminal portion, rather than by sharing a common function [6]. The human IAP family is composed of eight members: NAIP, cIAP1, cIAP2, XIAP, Survivin, Bruce/Apollon, ML-IAP/Livin and ILP-2, although XIAP, cIAP1 and cIAP2 are the most extensively studied [7,8,9,10]. First identified in viruses, they received the name of inhibitor of apoptosis proteins due to the function they exert on host cells [11]. In this regard, the process can be regulated both directly on caspases, through the blockage of substrate entry and ubiquitin-dependent mechanisms, or in an indirect manner through the regulation of nuclear factor kappa-light-chain-enhancer of activated B cell (NF-κB) canonical and non-canonical signaling cascades [12]. In addition to BIR domains, this function as an inhibitor of apoptosis is supported by the presence of various other domains which are not conserved among the different family members. For that reason, it is not surprising that IAPs have additional functions, participating in other processes, such as cell-cycle regulation or cell migration [13,14,15]. In this regard, the multiplicity of pathways in which IAPs participate and, most importantly, their abnormal function, due to dysregulation in IAP expression, may add to the pathogenesis of diseases, such as cancer. Importantly, in these pathologies, including breast, colorectal and hematological cancers, the eight identified members are often overexpressed. As outlined above, cancers are not the only pathologies affected by IAP dysregulation. In fact, different IAPs seem to play important roles in the correct function of the immune system. In this sense, aberrant expression of IAPs have been observed to be present in both primary and secondary immunodeficiencies and inflammasome-related pathologies [16].

On the other hand, second mitochondrial-derived activator of caspases (Smac), the second mitochondrial-derived activator of caspases, also known as DIABLO, is a mitochondrial protein released to the cytosol upon apoptotic stimuli that antagonizes IAPs endogenously, leading to the activation of caspases and the promotion of programmed cell death in normal conditions [17]. Given that in some cancers, Smac is downregulated along with its inherent IAP inhibitory structural properties, peptides mimicking its activity have been developed. These compounds are commonly known as Smac mimetics and have been systematically tested during the past decade. In spite of promising pre-clinical results, further clinical trials evaluating the anticancer activity of Smac mimetics/IAP antagonists as a monotherapy failed to provide a homogeneous pattern of response. Instead, these studies showed a limited scope of action in only a subset of cancer types and patients. Additionally, Smac mimetics’ killing efficacy was suggested to be dependent on other molecules, such as interferons (IFNs) or tumor necrosis factor-α (TNF-α) [18]. This, again, pointed to the well-established complex nature of IAPs’ biology, emphasized by the fact that the inhibition of apoptosis seems to be only one of this class of protein’s many functions. Despite all that, when tested in combination with different anticancer agents, Smac mimetic-based therapy showed synergizing effects and led to remission in many cases [19,20].

Recently, some investigations have reinforced the fact that these cell death pathways (apoptosis, pyroptosis, and necroptosis) could interact with each other [21]. Pyroptosis is characterized by diminution of plasma membrane integrity and induced by activation of so-called inflammasome sensors, such as the Nod-like receptor (NLR) family, the Pyrin receptor, and DNA receptor Absent in Melanoma 2 [21,22], and necroptosis, which is a complementary type of regulated cell death, mimicking features of necrosis and apoptosis [23]. Indeed, the simultaneous engagement of three processes has been termed PANoptosis [22].

In this work, IAP structure, function, and role in cancer will be addressed, as well as anticancer therapies based on their inhibition to promote apoptosis. In addition, possible reasons for their modest effects, shall be presented incorporating new findings which may contribute to understanding of this phenomenon. Furthermore, the current state of combination therapy involving Smac mimetics with traditional agents as well as with immunotherapy, will be reviewed.

## 2. IAPs

The first human IAP was described in spinal muscular atrophy (SMA), when it was found to be partially deleted or mutated in type I SMA patients. Together with the causal *SMN* gene, NAIP was proposed to cause or contribute to SMA pathogenesis, which included dysregulated motor neuron apoptosis [24]. The following year, the following three human IAPs were identified: XIAP, cIAP1 and cIAP2 [25]. Soon after, survivin was identified in cancer and lymphoma, Bruce/Apollon in brain cancer, livin/ML-IAP as part of a homology search and ILP-2 in testis.

BIR domains are essential zinc-binding folds which mediate protein-protein interaction [26]. Consecutive BIR domains are suggested to increase affinity to their ligand and their binding properties are conserved in those molecules that harbor more than one [27]. XIAP, cIAP1 and cIAP2 possess three BIR domains. In these cases, BIR2 and BIR3 bind to IAP binding motif (IBM)-containing proteins, such as caspases, while BIR1 interacts with other signaling intermediates, such as TAB1 or TRAF1.

**Figure 1:**
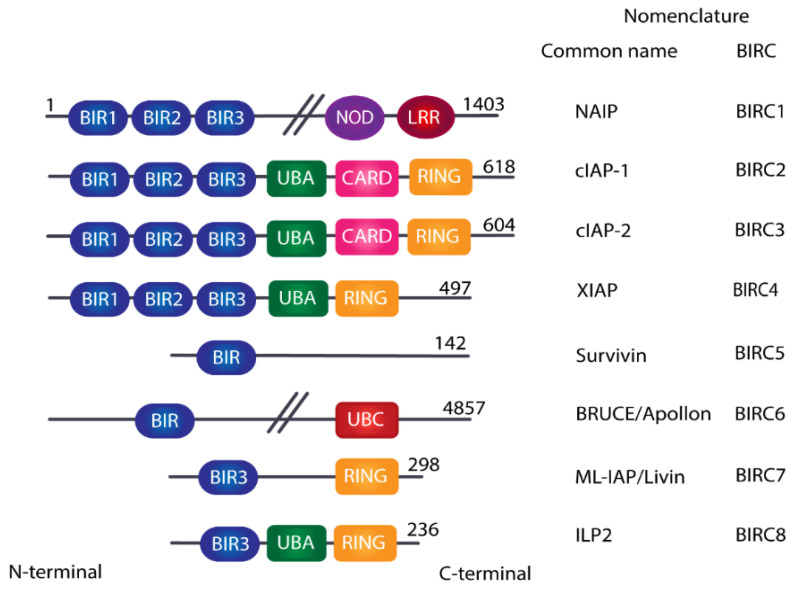
Schematic representation of the IAP family members’ structure. The nomenclature, length, and domains of the different proteins are shown. Abbreviations: BIR (Baculoviral IAP Repeat), NOD (Nucleotide-binding and Oligomerization Domain), LRR (Leucine Rich Repeat), UBA (Ubiquitin-Associated), CARD (Caspase-Associated Recruitment Domain), RING (Really Interesting New Gene), UBC (Ubiquitin-conjugating).

Additional domains are also observed in some IAPs (Figure 1). XIAP, cIAP-1 and cIAP-2 have RING (Really Interesting New Gene) domains. These contribute to ubiquitin-protein ligase (E3) activity which promotes the addition of ubiquitin residues to lysines or methionines on proteins [28]. CIAP-1 and c-IAP2 also possess a caspase-recruitment domain (CARD) which seems to participate in the regulation of E3 activity by preventing RING-mediated dimerization as well as E2 binding [14]. NAIP does not present CARD or RING domains. Instead, NAIP displays a nucleotide-binding oligomerization domain (NOD), which, together with the BIR domains, allows its participation in the inflammasome assembly in cooperation with NLRC4. NAIP also possesses a C-terminal leucine-rich (LRR) domain. Furthermore, other domains in IAPs are the evolutionarily conserved UBA (ubiquitin-associated) domains, which enable IAPs to bind to Lys-linked polyubiquitination [29].

### 2.1. Anti-Apoptotic Mechanisms

#### 2.1.1. Caspase Inhibition

IAPs participate in the inhibition of apoptosis through several mechanisms allowing their involvement in both extrinsic and intrinsic apoptotic programs. The first mechanism discovered was the inhibition of caspase activity [11]. However, under physiological conditions, IAPs are not enough to evade apoptosis, since the accumulation of apoptotic signals rapidly decides the cell’s fate. In turn, IAPs set an inhibitory threshold that caspases must overcome to result in cell death [30].

As caspase inhibitors, IAPs can reportedly either block substrate entry or add ubiquitin (Ub) chains on caspases targeting them for proteasomal degradation. According to Eckelman et al. [31], XIAP is the only direct caspase inhibitor in a strict biochemical sense since it inhibits their action through physical contact. Caspase-3 and -7 are blocked, as the linker region between the BIR1 and BIR2 domains of XIAP bind to the catalytic site of the cysteine proteases, preventing substrate entry. In the meantime, BIR2 can have contact with the IAP-binding motif (IBM) of the protein. Binding to both the catalytic pocket and the IBM results in an effective dock and inhibition of the effector caspases. Caspase 9 inhibition takes place when the BIR3 domain binds to the homodimerization surface of the cysteine protease, interfering with the production of a catalytic pocket [31].

CIAPs, on the other hand, inhibit caspases-3 and -7 by targeting them for proteasomal degradation through the addition of poly-Ub chains, by the action of their RING finger domains [32]. Knockout experiments in mice suggested overlapping activities of both cIAP1 and cIAP2, with cIAP1 having more biological impact. In this regard, later experiments showed viability in XIAP and cIAP1 null mice, with a marked upregulation of cIAP2 observed in double knockouts [33].

In addition to E3 ligase activity targeting other proteins for degradation, the three IAPs also share autodegradative capacity [34]. This function, observed in healthy cells, where RING bearing IAPs are downregulated, has been proposed to restrict levels of accidentally released IBM proteins by inducing co-degradation. This way explain normal phenotypes in mice deficient for these proteins [20]. However, in humans, defective expression of XIAP, due to deleterious mutations, is found to cause X-linked lymphoproliferative syndrome (XLP) type 2 [35,36].

Bruce/Apollon can modulate caspase-3, caspase-7 and caspase-9. For instance, it can polyubiquitylate caspase-9, when ectopically expressed in vitro [37,38,39].

NAIP is capable of inhibiting effector caspases-3 and -7. Interestingly, it can also process caspase-9 once it is in the apoptosome complex, through the action of the BIR3 domain in a unique way, different from the other IAPs’ modes of action [40].

Both survivin and livin are normally expressed during fetal development and not in adult tissues [41]. Consistent with its cell cycle-dependent expression, survivin is translocated from the cytoplasm to the nucleus during the G2/M phase. It is believed that the E3 activity of both survivin’s and livin’s RING domain might be responsible for their subcellular localization [42]. As for their anti-apoptotic activities, survivin and livin contain one BIR domain that interacts with both caspase-3 and -7, regarding the former, and with caspase-3, -7 and -9, respectively [15].

In addition to direct function as caspase inhibition, the IAP family modulates apoptotic events modulating the signaling cascade of NF-κB pathways leading to cell survival. These pathways are associated with innate and adaptive immune responses, cell survival, proliferation, growth, or motility, depending on the cell type.

#### 2.1.2. Regulation of Survival Pathways

The family of transcription factors known as NF-κB comprises five different proteins belonging to the Rel Homology Domain (RHD)-containing family: RelA (p65), RelB, c-Rel, p105/50 (NF-kB1) and p100/52 (NF-kB1). Only three of the members (RelA, RelB and c-Rel) are produced in their mature forms, while the other two (NF-kB1 and NF-kB2) require a proteolytic cleavage of the precursor’s C-terminal portion. These later molecules do not contain transactivation domains (TAD), which are required for the activation of NF-κB genes; so, they rely on interaction with the other members. In order to become active, different members combine to form dimers. Different signals leading to NF-κB formation combined with different dimer combinations account for the modulation of the different NF-κB transcriptional programs. The two main signaling cascades that activate NF-κB can be classified either as classical (canonical) or alternative (non-canonical) pathways. Importantly, both processes are governed by the addition or deletion of Ub residues on complexes that lead to the activation of the kinases controlling NF-κB activation [43].

The NF-κB canonical pathway is classically activated upon stimulation of TNFR1, IL-1E1, TLRs, BCR or TCR. It depends on the assembly of complex I and relies on the phosphorylation of IkBα (NF-κB inhibitor) by IKK. Complex I, composed of TNFR1, TRADD, RIPK1, TRAF2 and cIAP1 is formed upon TNF-α binding to TNFR. CIAP1 ubiquitylates many components of the complex amongst which RIPK1 is essential. Once ubiquitylated, these components recruit LUBAC, TAK1-TAB2-TAB3 and IKKγ-IKKα-IKKβ. LUBAC, in turn, conjugates M1-linked Ub chains onto IKK which stabilizes the complex. IKK then phosphorylates NF-kB1β, targeting it for proteasomal degradation. NF-κB (p50p65) translocates to the nucleus and transcribes survival and inflammation-related genes. In addition, it promotes the expression of cIAP1, cIAP2, and XIAP. In the absence of IAPs, complex II is formed. This new complex is derived from complex I when it detaches from the receptor. The formation of complex II is dependent on RIPK1 and leads to the recruitment and activation of caspase-8, leading to apoptosis [44].

The NF-κB alternative pathway can be initiated by the stimulation of BAFFR, CD40, HTLV or EBV and activates non-canonical NF-κB complexes. In resting conditions, cIAPs target NIK for ubiquitylation and proteasomal degradation. This is possible through the action of TRAF2 and TRAF3. However, upon substrate binding after recognition of CD40L by CD40, the ligand-receptor complex recruits TRAF3-TRAF2-cIAP for ubiquitylation of TRAF3. Ubiquitylated TRAF3 undergoes proteasomal degradation impeding NIK elimination. Accumulation of NIK activates IKKα which, in turn, phosphorylates NF-κB 2 (p100), transforming it into p52 and allowing nuclear translocation of the p52-RelB heterodimer [45,46].

**Figure 2:**
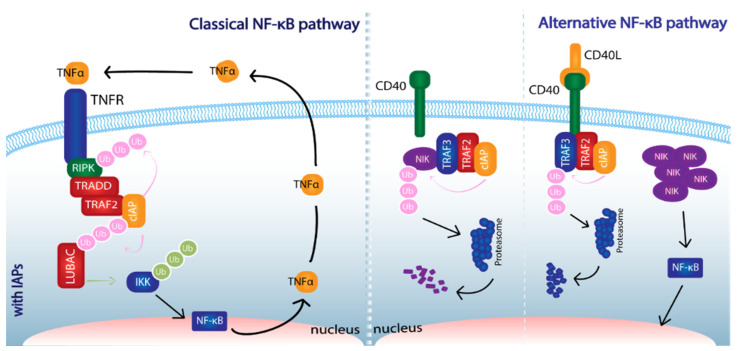
Participation of cIAPs in NF-κB classical and alternative pathways. E3 ligase activity from IAPs’ RING domain allow the addition of Ub residues onto proteins involved in these signaling cascades. In the classical pathway, cIAPs have a positive regulatory function while they exhibit negative regulation in the alternative pathway. Abbreviations: NF-κB (nuclear factor kappa-light-chain-enhancer of activated B cells), TNF-α (tumor necrosis factor-α), RIPK (Receptor-interacting serine/threonine-protein kinase), TRADD (TNF Receptor Associated Death Domain), TRAF (TNF Receptor Associated Factor), cIAP (cellular-Inhibitor of Apoptosis Protein), LUBAC (Linear Ubiquitination Assembly Complex), IKK (IκB Kinase), NIK (NF-κB-inducing kinase), Ub (ubiquitin).

In such a way, cIAPs exert both positive and negative regulation on NF-κB activation (Figure 2). Therefore, in cells are deficient for cIAPs, the classical NF-κB pathway is proapoptotic, halting the expression of target genes, including IAPs or inflammatory cytokines. In this scenario, the positive feedback loop, in which the production of IAPs and the NF-κB pathway participate, stops. However, in these same conditions, the alternative pathway is activated, due to lack of destruction of NIK, which allows activation of non-canonical NF-κB complexes. Since constitutive NF-κB activation and promoted inflammation have critical roles in tumor development, and have been observed in several cancer types, the regulation of said pathways has been suggested as a therapeutical approach. Along these lines, realization that IAPs play critical roles in the modulation of said pathways opens new doors in terms of new access points to their regulation.

## 3. IAPs in Cancer

Traditional cancer chemotherapies are frequently based on the induction of apoptosis in cancerous cells. Many molecular mechanisms can be involved in resistance to chemotherapeutics, amongst which the evasion of apoptosis is frequently observed. Indeed, the avoidance of programmed cell death is a characteristic hallmark in cancers. Dysregulation in pathways that modulate apoptosis-like JNK, which affects p53 expression, or Notch-1, is found to promote this effect. Mutations on genes coding proteins involved in the apoptotic process, or overexpression of pro-apoptotic proteins, are also common [47]. Consistently, observation that IAPs are overexpressed in many hematological and solid cancers suggests their direct or indirect participation in their refractory nature. Also, different levels of IAPs’ expression in distinct patient groups have hinted at a possible involvement in treatment response and have been suggested to possess great potential as prognostic markers. As a result, new therapies targeting IAPs’ expression have been designed to re-sensitize cancerous cells to apoptosis. The latest evidence supporting the role of IAPs in some death-resistant cancers and cancer progression are reviewed next.

### 3.1. Acute Myeloid Leukemia (AML)

Studies evaluating the role of different IAPs in AM have revealed heterogeneous patient profiles while analyses of IAPs’ expression have failed to provide strong evidence that establishes IAPs as potent individual prognostic markers. In the early 2000s, two studies found a correlation between lower levels of XIAP protein with longer survival [48,49]. However, a third study showed no correlation between the levels of XIAP protein expression and survival was found [50]. In 2007, Hess et al. [51], analyzed pro- and anti-apoptotic gene expression in AML patients, documenting a three-gene, (including BIRC3 (c-IAP2), expression signature associated with poor overall survival (OS) [51]. High levels of ML-IAP/Livin protein have also been correlated with poor OS [52]. A later study, by Pluta et al. [53], evaluated correlations both between XIAP, c-IAP1, c-IAP2 and survivin with each other and with Smac/Diablo in newly diagnosed AML patients. Although XIAP showed strong correlations with c-IAP1 and c-IAP2 and the latter two with each other, no correlation with survivin was observed. Low levels of IAPs were associated with 100% complete remission (CR), while high levels of either one, two or three IAPs significantly reduced the percentage of patients attaining CR. On the other hand, a short OS was influenced by older age (>50), poor risk karyotype, low levels of Smac/DIABLO and high levels of survivin [53]. Along these lines, survivin expression was observed in 98% of the samples and protein and mRNA levels were shown to be higher than any other IAP measured in these patients. Taken together, results from Tamm et al. [48], Carter et al. [50], and Pluta et al. [53], appear to suggest an influence of survivin in OS that is stronger than that of XIAP, although further studies assessing how the presence of individual IAPs affect treatment and CR achievement are clearly required [48,50,53].

Importantly, different authors failed to find similar results when studying younger AML patients. Indeed, despite the classical assumption that childhood cancers are similar to adult ones, evidence against this model is becoming more obvious. Childhood AML was recently defined as a significantly different disease from adult AML at the genetic level [54]. However, genes encoding IAPs were not included in the genomic analysis performed on the infant cohorts as part of the Therapeutically Applicable Research to Generate Effective Treatments (TARGET) initiative. Therefore, over time, more and more studies evaluating childhood malignancies as independent diseases have started to appear. Along these lines, some studies have assessed the role of IAPs’ expression on survival and therapeutic response in children. Over the past decade, high levels of XIAP were found to positively correlate with risk groups, worse response to chemotherapy and shorter overall and relapse-free survival [49]. Livin and survivin levels were recently analyzed in two AML subtypes, acute promyelocytic (APL) and non-promyelocytic (non-APL). Survivin-negative patients were observed to exhibit longer survival independently of the AML group. On the other hand, livin negative patients presented longer survivals but only in the APL group. Furthermore, risk of relapse was associated with higher expression of survivin but not livin, although both correlated with higher levels of primary WBC. Taken together, none of them were identified as independent prognostic factors, although associations with other AML poor prognostic factors were evident [55]. While these findings agree with those of Makhlouf et al. [56] and El-Mesallamy et al. [52], they stand in strong contrast to the results presented by Sung et al., Carter et al, Moore et al. or Ibrahim et al. [57,58,59,60].

The overexpression of apollon was correlated with an unfavorable prognosis in pediatric patients, a finding first observed by Sung et al. [60] and later confirmed by Ismail et al. [61]. In both studies, apollon was identified as a promising prognostic factor due to its association with a higher risk of relapse, worse response to chemotherapy and decreased survival. Indeed, cutoff values for the expression of the BIRC6 gene were observed to be potent prognostic metrics for both poor clinical outcomes and worse response to therapy [60,61].

All in all, although the cited studies point towards associations between IAPs and AML, larger cohorts, stratification by relevant factors such as disease subtype, cytogenetic characteristics or age, and unified methodology must be analyzed in future studies. Additionally, data obtained from mRNA levels must be assessed and compared to those of protein in both adult and child populations in order to validate these IAPs as prognostic factors.

### 3.2. Chronic Lymphocytic Leukemia (CLL)

Chronic lymphocytic leukemia is the most common leukemia in adults. CLL slowly develops for extended periods of time before showing any symptoms, allowing patients to have normal lives during the early stages. However, in contrast to acute leukemias, which develop much faster, it is harder to cure. CLL is a heterogeneous disease characterized by the accumulation of monoclonal mature CD5+ B cells in lymphoid organs, bone marrow and peripheral blood. The accumulation of CLL cells has classically been suggested to be caused by the inhibition of spontaneous apoptosis, rather than by cell proliferation. In turn, given CLL cells fail to engage apoptotic programs in vivo although not in vitro, as observed by Collin et al. [62], it becomes apparent that the tumor microenvironment might have a role in these differences [62]. In fact, spontaneous apoptosis has been observed to be prohibited in patients with progressive disease compared to those with stable disease, which also correlated with higher expression of cIAP1, cIAP2, XIAP and survivin [63]. Lower Smac/DIABLO protein levels also correlated with progressive disease. Additionally, Grzybowska et al. [63] found the co-expression of survivin and cIAP1to be a negative prognostic factor, since patients presenting it showed shorter OS [63].

Despite the classical definition of CLL as a non-proliferative disease, some studies have paid attention to the proliferative nature of CLL cells found in ‘pseudo-follicles’ formed in lymphoid organs [64]. These cells are known to receive support from accessory cells, such as monocyte-derived nurse-like cells, follicular dendritic cells, BMSCs or T lymphocytes, which provide pro-survival and proliferative signals. Indeed, several pro-survival signaling pathways, including NF-kB, are known to be constitutively activated in CLL [65]. It is believed that such a microenvironment is accountable for resistance to chemotherapeutics. Growing evidence indicates that CLL proliferative cells might be responsible for disease relapse after treatment, so any approach targeting said microenvironment could mean a complete clear out of malignant cells [66]. Purroy et al. [67] sensitized resistant CLL cells to cytotoxic agents by inhibiting survivin expression and inducing apoptosis in an ex vivo model of this microenvironment [67]. Likewise, Zhu et al. [68] achieved sensitization of CLL cells to chemotherapy by modulating the expression of XIAP, among other antiapoptotic proteins, with microRNAs shown to be downregulated in ‘p53 wild-type’ patients from a Chinese cohort [68].

### 3.3. Colorectal Carcinoma

Colorectal cancer (CRC), also known as bowel cancer, was the third most diagnosed cancer, and the second most fatal, worldwide in 2018, according to “Las cifras del cancer” report from the SEOM (Sociedad Española de Oncología Médica). Although it is mostly a curable disease, when detected at early stages, when symptoms arise, cancer may already have advanced. Lack of activity, obesity, high-fat diet as well as alcohol and smoking are all risk factors for sporadic CRC. Altered molecular pathways documented in CRC have delineated a large number of potential biomarkers. However, given that current screening methods present specificity, sensitivity and cost limitations, make it has proven difficult to rapidly advance towards personalized and targeted therapies. Additionally, studies aiming for validation of these markers show small sample sizes, have data interpretation limitations and lack standard methodologies causing reproducibility challenges [69]. Despite these barrier, promising advances are being made. EGFR-targeted therapies use the KRAS gene as a predictive marker of response, and anti-VEGF is sometimes used in metastatic disease in combination with chemotherapy [69].

Survivin and cIAP-2 expression were distinctive in elderly groups with CRC compared to younger patients, which suggested age-related differences in CRC. Interestingly, XIAP expression was comparable in normal and cancerous tissues in both young and older patients [70]. The location of both cIAP-1 and cIAP-2 was assessed by Ponnelle et al. [71] who found that, although the proteins were present in both nucleus and cytoplasm, cIAP-1 was most frequently expressed in the nucleus (85%) and cIAP-2 in the cytoplasm (82%) in CRC samples [71]. However, nuclear expression of cIAP-2 was associated with lymphoid infiltrate in the stroma, suggesting a nuclear localization role of both proteins in the pathogenesis of CRC. Similarly, Karasawa et al. [72] observed higher cIAP-2 expression in stage II CRC with lymph node metastasis [72]. These patients also presented early recurrence after fluorouracil-based chemotherapy. Interestingly, lymph node metastases with low levels of cIAP-2 and TUCAN (CARD8) showed a larger percentage of five-year survival in a 2005 study of IAPs’ expression [73].

Correlation between XIAP expression and clinical features or prognosis in CRC have yielded conflicting results with some studies reporting no differential expression between cancerous and surrounding normal tissues, while others observed both higher protein and mRNA expression in the former [70,74]. Guoan et al. [75] showed XIAP staining with immunocytochemistry in the cytoplasm of CRC cells [75]. Also, a significant correlation between higher levels of XIAP and tumor differentiation, venous invasion and Duke’s staging was found. Furthermore, XIAP expression levels varied inversely with different survival indexes, such as PFS or OS, in most studies [74]. Takeuchi et al. [76] described the Akt signaling pathway as an important cascade through which XIAP appeared to be upregulated in C-Met overexpressing CRC cells [76].

Interestingly, livin expression significantly correlated with longer survival in a study by Lee et al. [74] in contrast to what was observed for XIAP, while Takeuchi et al. [76] did not find mRNA or protein overexpression in malignant cells [74,76].

BRUCE/Apollon mRNA was also found to be overexpressed in CRC tissues compared with non-neoplastic samples through cDNA microarrays [77] correlating with unfavorable clinical features. Smac/DIABLO levels have also been evaluated with respect to their prognostic potential in CRC and one study reported its decreased expression as an independent factor for poor prognosis [78].

### 3.4. Breast Carcinoma

In 2020, breast cancer was the most commonly diagnosed cancer worldwide, surpassing lung cancer. It is the most common cancer among women and the second most common cause of mortality in this population. However, the establishment of prevention programs, although still challenging, has allowed an increase in early detection and survival of patients [79].

In the last few decades, the discovery of hormonal alterations in breast cancer patients and the application of targeted therapies in populations with specific molecular characteristics have brought therapeutic promise. In terms of IAPs, overexpression in cancerous tissues has been generally observed in comparison to normal tissues. A trend of increasing expression in more advanced diseases is also apparent. Pluta et al., [80] evaluated XIAP, cIAP1, cIAP2 and survivin expression in breast cancer samples at diagnosis. The expression of XIAP and survivin was higher in more advanced cancers. Expression of cIAP-2 was higher in node-positive breast cancer, although cIAP-1 levels did not correlate with clinicopathological features of this malignancy. Higher expression of XIAP was found in most patients compared to control. Its overexpression was also associated with advanced cancer [80]. Yang et al. [81], observed an increase in XIAP expression in higher grades of ductal invasive breast carcinoma and ductal breast carcinoma in situ [81]. Similarly, immunohistochemical assays confirmed its expression in 84% of breast invasive ductal carcinomas with high immunoscores [82]. The distinction between cytoplasmic and nuclear localization has also been made. Zhang et al. [82], reported differential subcellular expressions in breast cancer samples when cytoplasmic XIAP was observed in all the studied cases but nuclear staining in only 43% [82]. Likewise, Xu et al. [83], found higher cytoplasmic expression compared to normal tissues, while nuclear expression showed no difference [83]. Cytoplasmic localization correlated to HER-2 expression status, and mutant-type (P53) status, and was considered to be a prognostic biomarker for basal-like breast cancer. Patients with higher tumor levels of XIAP showed an increased risk of relapse, as observed in database analysis. Zhang et al. [82], reported a significant relationship between nuclear staining of XIAP protein and shorter OS while Pluta et al. [80], observed associations between shorter PFS and XIAP protein expression [80,82]. However, Xu et al. [83], did not find a correlation with DFS or OS [83]. Tumor size, extranodal extension, triple-negative status and poorly differentiated subtypes showed direct associations with XIAP expression in the Middle Eastern population. This study showed a significant relationship with the PI3K pathway, p-AKT, Ki-67 and PARP. XIAP was proven to be an independent prognostic marker [84].

Similar to XIAP expression, survivin expression has been correlated with worse clinicopathological features, such as metastasis, and advanced stage, or size, of the tumor. In addition, ER and progesterone receptor-negative hormonal status was observed in to correlate with survivin level [85]. However, previous studies had not found any correlation with said features [86]. In particular, Adamkov et al. [87], suggested that the disagreement observed between different studies could have an origin in the subcellular localization of survivin [87]. Their experiments led to the determination of survivin expression as a potential prognostic factor for ductal breast carcinoma. A meta-analysis encompassing 15 studies, with a total of 2202 breast cancer patients, confirmed significant associations between positive expression of survivin and worse OS [88].

High expression of livin has been associated with highly-invasive breast cancer cells in opposition to a lower expression in non-invasive cells [13]. Li et al. [13], described the role of livin in cancer cell migration and invasion by means of the activation of Akt signaling and the induction of EMT in vitro and in vivo [13]. Furthermore, as in other malignancies, Smac/DIABLO protein was observed to be lowered in breast cancer samples compared to control [89].

Finally, despite the clear tendency of IAP expression to correlate with poor prognosis, Pluta et al. [80], who observed XIAP, cIAP1, cIAP2 and survivin expression in breast cancer samples at diagnosis, its effect on survival could not be confirmed [80]. IAPs’ patterns of expression in other malignancies, such as bladder, lung or renal cancers, are similar to those presented above and have been further developed by Fulda and Vucic [90] and Che et al. [91]. In glioblastomas, livin is correlated with worse PFS and OS rates. C-IAP1 and cIAP2 have been found to be overexpressed in osteosarcoma, and XIAP’s expression levels were high in esophageal, ovarian and pancreatic cancers [92,93]. Table 1 summarizes the IAPs and cancer.

## 4. Smac Mimetics

### 4.1. Smac/DIABLO

Smac, also known as DIABLO (Direct IAP Binding protein with Low pI), is a mitochondrial protein encoded in the nuclear genome. Although it is synthesized as a much longer precursor, in its mature form, it is composed of 184 amino acids. Upon induction of the intrinsic apoptotic pathway, mitochondria release Smac from their intermembrane space into the cytosol along with cytochrome C [17,94,95]. Apoptosis is a tightly regulated process in which the balance between different pro- and anti-apoptotic proteins decides the cell’s fate. Programmed cell death occurring through the intrinsic pathway is modulated by the Bcl-2 family of proteins, which govern mitochondrial membrane permeability [96]. Further down the pathway, IAPs have critical roles on caspase inhibition, setting an inhibitory threshold for these enzymes. Both IAPs’ direct caspase inhibition and poly-Ub-dependent caspase degradation can be easily overcome after massive release of IBM-containing proteins from mitochondria, including Smac/DIABLO. Dimeric Smac/DIABLO interacts with BIR2 and BIR3 IAP domains, ultimately leading to proteolytic cleavage and activation of procaspase-3 and procaspase-9, and the induction of caspase-3 enzymatic activity [97].

### 4.2. Smac Mimetics

Smac/DIABLO interaction with IAPs requires its first four N-terminal residues, also known as AVPI (Ala-Val-Pro-Ile). As previously outlined, Smac/DIABLO and IAPs regulate each other. IAPs auto-ubiquitylate when Smac/DIABLO induces their RING domain’s E3 activity. However, when overexpressed, IAPs can inhibit apoptosis as observed in cancers, contributing to the acquisition of resistance to first-line treatment traditionally based on inducing DNA damage. Inhibition of IAPs in experimental models with IAP overexpression achieved sensitization to apoptosis suggesting a potential therapeutic application [20,98,99,100,101]. Similarly, the use of exogenous Smac/DIABLO protein, both in vitro and in vivo, in several resistant cancer types, such as melanoma, neuroblastoma or glioblastoma, promoted the engagement of extrinsic and intrinsic apoptotic pathways [102]. However, Smac/DIABLO is a large protein that can have significant off target effects when delivered to patients at clinical levels. Therefore, compounds mimicking Smac/DIABLO’s antagonistic activity over IAPs have been developed in an effort to induce apoptosis in IAP-dependent apoptotic-resistant cells. The AVPI fragment, the minimal fragment conserving Smac’s inhibitory activity, has been systematically modified to optimize its pharmacological properties [103]. A total of four generations of these compounds, known as Smac mimetics, have been developed. GDC-0512, LCL16, SM-406/AT-406, Birinapant or ASTX660 stand out as promising compounds in current clinical evaluation.

#### 4.2.1. Smac Mimetics as Single Agents

Here, the development and effectiveness of the most clinically relevant Smac mimetics during pre-clinical experimentation will be described, as well as the results that preliminary investigation in the clinical contexts.

GDC-0152, is described as a pan-IAP antagonist because it has high affinities for cIAP-1, c-IAP2, XIAP-BIR3 and livin [104]. GDC-0152’s in vitro effectiveness was demonstrated on A2058 melanoma and different glioblastoma (GL261, U87MG, GBM6 and GBM9) cell lines, in which it successfully induced c-IAP degradation, activation of caspase-3 and -7 and apoptosis [93]. MDA-MB231 breast cancer xenograft mouse models benefited from antitumor activity in vivo when GDC-0152 was administered orally as a single agent. Also, U87MG orthotopic xenografts demonstrated delayed tumor growth upon administration of GDC-0152. The only trial assessing its safety and pharmacokinetics was terminated in 2017 due to patient or efficacy-unrelated matters (NCT00977067).

LCL161 is a peptidomimetic being evaluated in several clinical trials. It is orally available and capable of binding XIAP, c-IAP1 and c-IAP2 with IC_50_ of 52.7, 10.4 and 12.9 nM, respectively [105]. It was assessed in leukemia and hepatocellular carcinoma cell lines inducing cell death. Interestingly, in vitro studies with multiple myeloma cell lines observed resistance to LCL161, while in vivo they were sensitive to it [106,107,108]. Response to LCL161 was limited in 23 cell lines and 46 xenograft models of pediatric cancers [109]. A phase I study on solid tumors revealed good tolerability but poor efficacy [110]. Although the stable response was observed in 19% of patients, no objective response was observed. Additionally, the maximum tolerated dose (MTD) was determined at 1800 mg when higher doses provoked symptoms of cytokine release syndrome (CRS) in 6% of the patients: vomiting, fatigue, nausea and anorexia. However, cytokine levels, such as TNF-α augmented in circulation and cIAP1 degradation, were observed. Lack of tumoral TNF-α production and sensitivity was identified as a possible cause for the low efficacy obtained. On the other hand, an interesting thing was found in triple-negative breast cancer with a specific gene signature characterized by positive expression of TNF-α [111]. Promising results were observed when evaluated in combination with paclitaxel; although toxicity was highlighted as an important and limiting factor in the therapeutical scope.

Evidence of Birinapant’s efficacy has been modest but promising. According to Benetatos et al. [112], only 16% of malignancies were shown to be sensitive to Birinapant on a large-scale cellular screen. Human melanoma cell lines 451 Lu and 1025 Lu were resistant to Birinapant in vitro. Surprisingly, in vivo assessment of these same cell lines as xenotransplants showed their decreased growth. Similarly, one-third of the 50 patient-derived xenotransplants of ovarian, colorectal and melanoma cell lines studied acquired growth inhibition. Again, such differences between in vivo and in vitro results were attributed to higher expression of TNF in the tumor’s microenvironment [112]. Clinical evaluation of this compound was initially on advanced solid tumors or lymphoma (NCT00993239), where MTD was established at 47 mg/m^2^. Adverse effects were evident for higher doses and comprised headaches, nausea, vomiting and Bell’s palsy in 2 of the 3 patients receiving the highest dose. Similar to LCL161, no complete or partial response was observed in patients, although stable disease was achieved in 27%. In a different study for relapsed AML or MDS, one case of Bell’s palsy was also observed but, at the same time, there was a reduction of blasts in the bone marrow. Moreover, in a relapsed platinum-resistant, or refractory epithelial ovarian cancer phase II trial (NCT01681368) no clinical benefit was observed and therefore, the trial was terminated, despite observation of potent on-target inhibition of IAPs. Moreover, a trial assessing its efficacy on advanced ovarian, fallopian tube and peritoneal cancer had to be canceled early due to lack of clinical benefit (NCT01681368). Similarly, NCT01486784, evaluating Birinapant in AML, ALL and Myelodysplasic Syndrome never initiated its phase II portion, due to early termination. All in all, Birinapant showed moderate antitumoral activity, but efficacy was far from ideal.

Debio 1143/AT-406 was firstly described in 2011 shown to mimic AVPI’s binding to BIR3. It induces cIAP1 degradation and binds to XIAP and cIAP-2. Researchers assessed this compound effectiveness as a single agent in more than 100 human cancer cell lines, although only 15% of the cell lines were responsive to the treatment, MDA-MB-231 breast and SK-OV-3 ovarian cancer lines demonstrated high levels of cell death. Furthermore, xenograft models of MDA-MB-231 cell line had efficient induction of caspase-8 dependent apoptosis with a reduction in cIAP1 levels [113]. In the first-in-man study evaluating Debbio1143 in advanced cancer, results were very modest and were not able to shed light on a recommendable dose to work with in further studies despite its good orally bioavailability. Very limited antitumor activity was hinted at, rather than observed, through the degradation of cIAP-1 [114]. Therefore, its use in combination with other therapies was proposed as a follow-up.

ASTX660 antagonizes cIAP-1, ciap-2 and XIAP through binding to BIR3. In vitro evaluation of its efficacy has demonstrated direct XIAP antagonism and depletion of cIAP-1 and cIAP-2 activating the alternative NF-κB pathway and leading to TNF-α dependent apoptosis in MDA-MB-231, HEK293, A375 and SK-MEL-28 cell lines, among others [115]. In this case, the first-in-human phase I study in advanced cancer or lymphoma succeeded in establishing an MTD of 210 mg/day. Severe adverse events included anemia, and increased lipase and lymphopenia, which were observed, but only in seven patients. As for its efficacy against IAPs, it successfully inhibited cIAP-1 in peripheral blood mononuclear cells and reduced skin lesions in patients with cutaneous T-cell lymphoma [116]. Phase II studies are still ongoing, but results obtained in the previous phase I are promising and support further evaluation (NCT02503423). Additionally, a recent clinical trial is currently recruiting participants to evaluate ASTX660′s safety and efficacy in patients with refractory or relapsing T-cell lymphoma (NCT04362007).

#### 4.2.2. The Role of TNF-α in the Efficacy of Smac Mimetics

**Figure 3:**
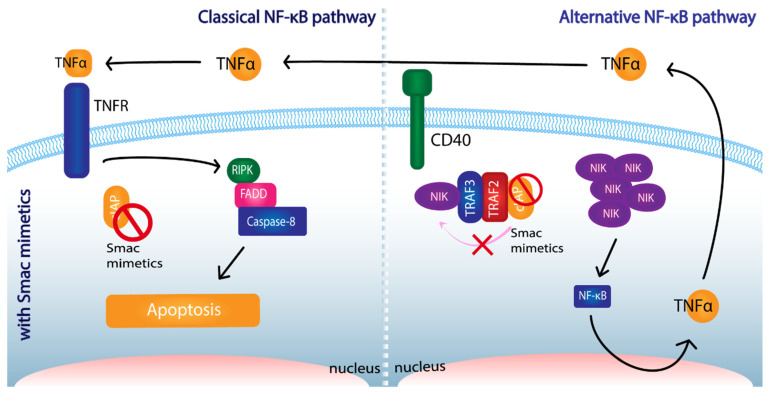
The role of Smac mimetics in the regulation of TNF-α dynamics and the promotion of apoptosis. The inhibition of cIAPs alters NF-κB pathways. The classical NF-κB pathway is pushed towards caspase-8 dependent apoptosis through the formation of RIPK-FADD-Caspase-8 complex and it is fed by the production of TNF-α in the alternative pathway. Abbreviations: TNF-α (Tumor Necrosis Factor-α), TNFR (Tumor Necrosis Factor Receptor), cIAP (cellular- Inhibitor of Apoptosis Protein), RIPK (Receptor-interacting serine/threonine-protein kinase), FADD (Fas-associated protein with death domain), TRAF (TNF receptor associated factor), NIK (NF-κB-inducing kinase), NF-κB (nuclear factor kappa-light-chain-enhancer of activated B cells).

The binding of TNF-α to TNFR can initiate the extrinsic apoptotic pathway. As outlined above, cIAPs have important roles in the regulation of the signaling cascade following the binding of TNF-α to its receptor. Several studies have revealed the importance of TNF-α in the effectiveness of Smac mimetics treatment. To sensitize cells to apoptosis, some cancers require production and response to TNF-α. For instance, LCL161 has been suggested to require the TNF-α pathway and TNF-α production to promote apoptosis [117]. Cancers showing no response to, or no expression of, TNF-α, have been observed to be resistant to Smac mimetics treatment in some circumstances. Therefore, some therapeutic approaches are based on the induction of TNF-α expression by tumor cells. One hypothesis argues that if tumors have access to TNF-α, the suppression of IAPs will halt the NF-κB classical pathway, re-routing it towards caspase-8 dependent apoptosis. At the same time, the lack of said cIAPs would allow the NF-κB alternative pathway to produce further TNF-α, which would feed an autocrine cycle leading to cell death (Figure 3).

Smac mimetics EAG40730 and ASTX660 have proven to induce cIAP autoubiquitination, allowing NIK accumulation and alternative NF-κB pathway activation. As a consequence, TNF-α provoked apoptosis [118]. Similar results have been obtained when assessing BV-6’s and Debbio 1143’s efficacies [119,120]. Hence, the administration of exogenous TNF-α has been evaluated. For safety reasons, due to the high toxicity of systemic delivery of TNF-α, targeted delivery methods had to be developed. The use of viral vectors and the induction of parallel pathways have been considered. In addition, radiation therapy appears to induce TNF-α production, and, in turn, TNF-α enhances radiation-mediated killing. Along these lines, the results obtained by Krepler et al. [121] are consistent with this role of TNF-α. They observed synergism between Birinapant and exogenously administered TNF-α on antitumoral activity over several melanoma cell lines [121]. Overall, Smac mimetics’ effects are generally understood to be enhanced by pro-inflammatory cytokines, such as TNF-α or TRAIL [46,122,123,124]. This phenomenon links IAPs to immunity, although it was not the first evidence of said relationship. This interesting association with immune activity will be further explored below.

Cancer showing resistance to chemotherapy, which is the first line of treatment for many cancer types, has also shown dysregulation in IAP expression. Therefore, inhibition of IAPs is being trialed in combination with chemotherapy. However, some cancers can be both responsive and non-responsive to Smac mimetics, irrespective of their TNF-α status. For instance, NSCLC cell lines were sensitized to chemotherapy by the SM JP1201 even when TNF-α expression was not high [125]. Later, Langemann et al. [126], showed that the effect of LCL161 on vincristine-induced neuroblastoma apoptosis was independent of NF-κB and TNF-α [126]. Similarly, Birinapant efficacy was observed to be TNF-α independent in SUM149 and SUM190 inflammatory breast cancer cell lines [127].

In addition to apoptosis, necrosis can also be promoted by IAPs. It can start from TNFR1 signaling, which can induce both apoptosis and necroptosis. In some cells, alterations in the caspase-8-dependent apoptotic pathway, provoked by caspase inhibitors or genetic defects, can switch the cell death to necrosis, depending on the levels of RIP3. BV6 and Birinapant are both capable of inducing TNF-induced cell death. Before being used as clinical agents, these Smac mimetics were used in the study of necrosis [83]. Indeed, according to Yabal et al. [128], XIAP protein might be involved in RIP1 and RIP3 ubiquitylation in the necrosome but, instead of promoting it, they regulate it, since deletion of XIAP renders elevated RIP1 ubiquitylation [128]. Apoptotic/necrotic death induced by Smac mimetics and TNF-α crucially depends on RIPK1 because RIPK1 deficient MEFs, and cancer cells in which RIPK1 is depleted, are resistant to TNFR1 killing following treatment with Smac mimetics [46,123]. Finally, other pathways leading to apoptosis, such as that derived from endoplasmic reticulum (ER) stress induction, appear to be, in part, modulated by the presence of IAPs too. Thus, Smac mimetics treatment might exert its function in a wider spectrum of signaling pathways than previously anticipated [129].

#### 4.2.3. The Role of Endoplasmic Reticulum (ER) Stress in IAP Inhibition

In cancer, different factors, including hypoxia or genomic instability, increase the demand for protein production, resulting in accumulation of proteins, which end up promoting ER stress [130]. Likewise, ER-stress-derived-NF-κB activation, and consequent inflammation, have been linked to numerous cancer types.

During the last decades, the Unfolded Protein Response (UPR) to ER stress has been systematically studied and associated with cancer pathogenesis [131]. It is composed of three effector mechanisms that can lead to adaptation, survival or apoptosis, respectively, and depends on stimulus and cell type. Furthermore, although it is normally started as a response to ER stress to alleviate potential proteotoxicities, its pro-survival capacity can contribute to tumorigenesis and cancer progression [132,133]. For instance, ER stress has been described as participating in the acquisition of drug resistance in AML, where ER stress appears to be transmitted through extracellular vesicles among leukemic and bone marrow resident cells. This phenomenon forces the remodeling of the bone marrow niche contributing to AML expansion and is potentially involved in drug resistance [129]. In addition, the engagement of the UPR adaptive response to ER stress is known to provoke drug resistance in other malignancies, such as pancreatic and breast cancers.

Interestingly, among the different pathways elicited as part of the pro-survival UPR, some have been observed to somewhat regulate IAPs’ expression [134]. Nevertheless, while adaptive UPR has been shown to upregulate cIAP1, cIAP2, and XIAP through PERK (one of the sensors of the UPR), chronic ER stress was observed to downregulate XIAP protein levels, promoting a switch towards the apoptotic mechanism [134]. This downregulation is also mediated by PERK, which halts global protein translation after activation, and the activation of ATF4. Additionally, Hu et al. [135], demonstrated that ER stress might upregulate IAPs in different cancer cells through the PI3K/Akt pathway, which could be elicited by Ca^2+^ imbalances normally found in such conditions [135]. The ablation of IAPs in Hu and colleagues’ experiments did not completely decrease cell survival, indicating the contribution of other pathways in IAP upregulation. However, there is limited information regarding the contribution of both mechanisms to actual increases in IAP expression in specific cancer types. Therefore, further research is required to elucidate the contribution of each mechanism to both apoptosis and therapy resistance. Altogether, available data suggest a strong link between IAP-mediated apoptosis inhibition and ER stress induction, although it remains unclear whether the UPR apoptosis program contributes or not (and to what extent) to cell death promoted by Smac mimetics treatment [135]. In this regard, it should be noted that the evaluation of the Smac mimetic JP1202 on NSCLC cell line described TNF-α independent pathways as being responsible for cell death induced by the treatment. The researchers highlighted the participation of ER stress as observed by the activation of caspase-4 [125].

## 5. Combination Therapy

Cells with dysregulated apoptosis cannot induce cell death; they continue dividing. Upon IAPs’ downregulation by antisense oligonucleotides both in vitro and in vivo, pre-clinical resistant cell models are able to engage in the apoptotic programs [36]. As already stated, IAPs’ inhibition with Smac mimetics has demonstrated some clinical relevance, although their effectiveness has been limited when assessed as individual agents. In parallel, some chemotherapeutic approaches, despite being the main, or even the only, therapy for some cancers, have modest efficacy. For example, this is the case with NSCLC, where paclitaxel only shows modest efficacy and data is limited [136]. Consequently, combination approaches have been evaluated. The effect of LCL161 on NSCLC cell lines was studied in combination with paclitaxel, where increased expression of TNF-α accompanied by degradation of cIAPs, were observed to precede caspase-8 dependent cell death in vitro [136]. Xenograft models indicated similar results, where the combination therapy worked better than any of the two as single agents. Taking into account TNF-α expression as an important factor in antitumoral response, a clinical study on triple-negative breast cancer (TNBC) used TNF-α expression profiles as a biomarker to evaluate patients’ response to LCL161 and paclitaxel. Promising results showed a possible correlation between clinical efficacy and TNF-α positive TNBC patients with both LCL161 and paclitaxel. However, due to higher discontinuation provoked by AEs in the TNF-α negative TNBC patient group, lower efficacy observed could be attributed to either the consequent lower paclitaxel exposure or their TNF-α status [111]. The authors were therefore prevented from giving a more concrete opinion about the role of TNF-α in TNBC. Birinapant has been assayed with several chemotherapeutics due to its good tolerability. In vitro studies have revealed higher efficacy in combination with chemotherapy than that of any of the agents alone [137]. Combination with docetaxel achieved tumor volume reduction in HNSCC xenograft models, although survival was not affected. Carboplatin and Birinapant sensitized cells to caspase-8 dependent killing in High-Grade Serous Ovarian Cancer in both in vitro and in vivo models [138]. Interestingly, combination with gemcitabine showed no involvement of TNF-dependent death mechanisms. Debio 1143 is capable of synergizing with taxanes, among other agents, against lung adenocarcinoma cells, even reducing the tumor volume in mice xenografts [139]. A phase I/II trial of Debio in combination with cisplatin in patients with locally advanced squamous cell carcinoma of the head and neck provided encouraging results including high OS, CR and PFS rates [140]. Furthermore, it demonstrated efficacy in mice models of carboplatin-resistant ovarian cancer in combination with carboplatin, which is currently the first line of treatment, despite eventual relapse. Survival was improved and correlated with XIAP downregulation [141].

Other therapeutical approaches have been assessed in combination with Smac mimetics including TRAIL, radiation and monoclonal antibodies have been assayed together with different Smac mimetics. Importantly, and given the recent evidence of IAPs’ role in the immune system, different immunotherapeutic approaches have been suggested to potentially benefit from Smac mimetics’ co-treatment. Immunotherapy-based treatment harnesses the immune system to fight cancerous cells. On their own, immunotherapy approaches can show impressive efficacy. Despite limited effects on large patient cohorts and adverse effects, as severe as induction of autoimmunity, cancers meeting very specific criteria seem highly sensitive to this approach. For example, immune checkpoint inhibitors (ICIs) have already succeeded in stimulating T cell responses against tumors. Nevertheless, an important observation was made by Kearney et al. [142], when PD-L1 expression seemed to inhibit TNF-α production in cytotoxic lymphocytes, avoiding a possible paracrine interaction with tumoral cells and the subsequent TNF-mediated cell death [142]. Interestingly, Birinapant was not able to increase said TNF-α levels, although it did sensitize tumor cells to TNF-mediated death induced by cytotoxic lymphocytes. These results pointed out the potential benefits of using ICIs together with Smac mimetics as a way of increasing TNF-αproduction, while forcing the TNFR pathway towards a caspase-8 dependent death. Accordingly a number of studies, proving and revising the role of IAPs on immune modulation through the activation of NF-κB pathways have been published [143].

Combination therapy of Smac mimetics with ICIs have provided good results in pre-clinical models. PD-1 and CLTA-4 extended survival rates of glioblastoma mouse models when administered in combination with LCL161 or Birinapant, despite not showing any activity on their own. Combined blockade with anti-PD1 and anti-CLTA-4 was assessed as in previous studies [144]. The addition of Smac mimetics to said combination led to a 100% durable cure rate. Interestingly, effective penetration of blood-brain barrier was achieved. Likewise, models of multiple myeloma and mammary tumors obtained very high percentages for durable care rates [145]. This effect was ascribed to the presence of cytotoxic T cells, and decrease in immunosuppressive CD4+ T cells, and seemed to involve IFNs and TNF-α pathways. Indeed, Bv6 was found to synergize with IFN-γ in the promotion of necroptosis in a TNF-α independent manner [18].

The combination of Birinapant with several other therapies was being evaluated but different trials had to be terminated early due to Birinapant’s lack of clinical benefit (NCT02147873), (NCT01681368). Similarly, a trial assessing its combination with pembrolizumab in solid tumors (colorectal, ovarian and cervical carcinomas, among others) had to be canceled, based on futility analysis (NCT02587962). LCL161 evaluation with an anti-PD1 monoclonal antibody is currently active in a phase I trial against colorectal cancer, TNBC, NSCLC and renal carcinoma (NCT02890069), and completed for multiple myeloma, although results are not yet available (NCT03111992).

Furthermore, the realization that Smac mimetics may have different roles on the immune system, apart from cIAPs’ involvement in NF-κB activation, has catalyzed different uses of these agents. INKT cells can be stimulated to produce cytokines upon Smac mimetics’ stimulation [146]. M2 macrophages can transform into M1 inflammatory macrophages, as observed in mice models of ovarian carcinoma and sarcoma cells. Inflammation-induced assayed Smac mimetics promoted the production of TNF-α, IL-1β and IFNγ, which supposedly provoked macrophage phenotypic change. The immune response, started as a result of SM83 administration (a bivalent Smac mimetics), terminated in necrotic death of cancerous cells [147]. According to Rigaud et al. [36], patients with XLP resulting from XIAP mutations were observed to have low numbers of natural killer T-cells (NKTs), which suggested a role for this IAP in lymphocyte homeostasis in vivo [36]. Also, CARD domains present in cIAPs appear to cooperate with c-Myc in proliferation/migration of cancers [14]. And, importantly, Smac mimetics have demonstrated effects on T cell cytokine secretion, enhanced in vitro priming of naïve T cells by peripheral blood mononuclear cells and dendritic cell maturation, suggesting possible roles in modulation of immune responses [148].

**Figure 4:**
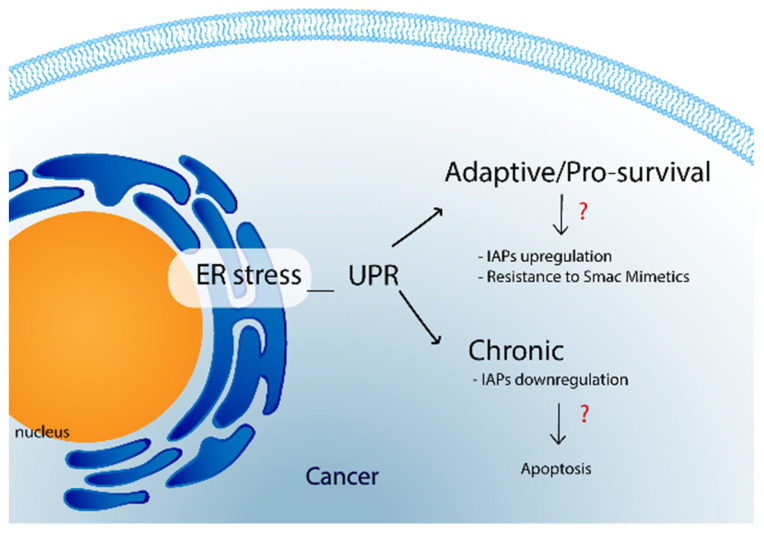
ER stress and Smac mimetics. Abbreviations, ER stress, endoplasmic reticulum stress; IAPs, inhibitor apoptosis proteins; UPR, unfolded protein response.

Lastly, given that both the enhancement and the inhibition of stress responses in cancer can achieve reduction in tumor growth, efforts in both directions have been made. The results of some of these approaches are collected in a review from Pakos-Zebrucka et. al. [149]. Despite the above, a direct link between ER stress and Smac mimetics’ resistance has not been established yet and, therefore, there is no information about the study of ER stress modulators and Smac mimetics combined therapy. However, there is evidence pointing towards a possible relationship between ER stress and Smac mimetics’ effectivity that cannot be ignored by future evaluations. Figure 4 summarizes that association.

## 6. Conclusions

IAPs play a crucial role in setting a threshold for cells to undergo programmed cell death upon apoptotic stimuli. The mechanisms through which these molecules exert their functions are varied, ranging from direct physical interaction with caspases to the regulation of cell survival pathways. In this sense, overexpression of IAPs has been observed to directly correlate with cancer progression and decreased survival. Therefore, therapeutic approaches based on mode action of IAPs’ endogenous antagonist, Smac/DIABLO, have been proposed. Smac mimetics are thus developed in an effort to decrease IAPs’ levels observed in cancers and other diseases in which their expression is dysregulated. In cancer, cells exhibiting upregulated IAPs are sometimes observed to show resistance to chemotherapy and other therapies based on the induction of apoptosis. Along these lines, reduction of IAP levels can potentially re-sensitize cells to apoptosis. This phenomenon is usually described as being enhanced by the presence of TNF-α, as TNF-α combined with the inhibition of IAPs achieves a switch in the TNFR-dependent NF-κB pathway towards caspase-8 dependent cell death. At the same time, NIK accumulation in NF-κB alternative pathway feeds a positive loop, allowing inflammation and antitumoral effect in some cases. However, and despite supporting evidence, in other cases, death promoted by administration of Smac mimetics is not dependent on TNF-α or NF-kB. The cause of such discrepancy is still unknown but further studies are needed to determine in which cancers the TNF-α status can or cannot be a reliable prognostic/response marker. In parallel, different studies have demonstrated that ER stress can both induce and reduce the expression of IAPs. In addition, in some cancers, ER stress has been identified as responsible for the acquisition of resistance to chemotherapeutic agents. Together, a constitutive ER stress that promotes upregulation of IAPs could, at least in part, explain the loss of apoptotic capability. However, the total scope of the ER stress contribution on IAPs’ upregulation in cancer is not known, and neither are its connections with other routes also participating in IAPs’ overexpression. It might be worth studying the nature of Smac mimetics’ resistance. It would not be surprising to find that Smac mimetics’ resistance is not, at least in part, promoted by ER stress induction of IAPs’ overexpression, such that previously established doses of Smac mimetics might not be sufficient to overcome inhibition of apoptosis. This might be of particular interest in the context of TNF-α non-responsive cancers. Ultimately, a picture of multifactorial IAP overexpression and acquisition of resistance emerges. Therefore, it might be interesting to further investigate the pathways interacting to produce IAPs’ upregulation. Overall, this supports the evaluation of combination therapy in cancers, in which no therapeutic approach has provided positive results on its own.

Finally, not only apoptosis but also necrosis is promoted as part of Smac mimetics treatment, unpacking new layers of IAPs’ relationship with tumoral development/progression.

Furthermore, recent evidence demonstrating new immune-modulatory functions of IAPs, in both innate and adaptive immunity, opens new doors in terms of therapeutic opportunities. It is not only the inhibition of apoptosis, but also how these proteins interact with immune cells, that create a complex and fairly opaque mode of action and therefore make it difficult to predict Smac mimetics therapy outcomes. In this sense, further investigations should undertake, in terms of deciphering the complex web of interactions between Smac mimetics and inflammation, cell maturation and, in general, immune modulation for specific cancers. The elucidation of these converging routes would signify a huge advance in prognostic evaluation, which could be incorporated in pre-treatment screening to improve targeted therapies.

## Figures and Tables

**Table 1:** IAPs and cancer. Abbreviations: AML, acute myeloid leukemia; CLL, chronic lymphocytic leukemia; CRC, colorectal cancer; FU, fluorouracil; OS; overall survival.

Cancer Type	Observations	References
Acute Myeloid Leukemia (AML)		
XIAP	Lower levels of XIAP correlate with longer survival	Tamm et al. [48], Tamm et al. [49]
	No correlation between XIAP protein levels and survival	Carter et al. [50]
	XIAP expression strongly correlates with cIAP1 and cIAP2 but no correlation with survivin was found. Low IAPs levels point to higher complete remission rate.	Pluta et al. [53]
Survivin	Appear to influence on the OS and it might be stronger than that from XIAP expression.	Pluta et al. [53]
cIAP2	High gene expression of cIAP2 is associated with poor OS	El-Mesallamy et al. [52]
Livin	High level of Livin is correlated with poor OS	El-Mesallamy et al. [52]
**Childhood AML**		
XIAP	High levels of XIAP positively correlate with risk groups, worse response to chemotherapy and shorter OS.	Tamm et al. [49]
Livin	Livin negative patients showed longer OS in the acute promyelocytic AML	Zareifar et al. [55]
Survivin	Survivin negative patients showed longer OS	Zareifar et al. [55]
Apollon	Overexpression of apollon correlated with unfavorable prognosis. It was identified as a prognostic factor.	Ismail et al. [61], Sung et al. [60]
**Chronic Lymphocytic Leukemia**		
XIAP	Higher expression of XIAP, cIAP1, cIAP2 and survivin was observed in patients with progressive disease which also exhibited inhibition of spontaneous apoptosis.	Grzybowska-Izydorczyk et al. [63]
	Modulation of XIAP expression sensitized CLL cells to chemotherapy in humans.	Zhu et al. [68]
Survivin	Co-expression of survivin and cIAP1 was related with shorter OS and identified as a negative prognostic factor.	Grzybowska-Izydorczyk et al. [63]
	Inhibition of survivin sensitized CLL cells to cytotoxic agents and induced apoptosis in and *ex vivo* model.	Purroy et al. [67]
Smac	Lower levels of Smac protein correlated with progressive disease.	Grzybowska-Izydorczyk et al. [63]
**Colorectal Carcinoma (CRC)**		
Survivin	Survivin and cIAP2 expression was characteristic of elderly groups	Endo et al. [70]
XIAP	XIAP expression was comparable in both normal and cancerous tissue of old and young patients.	Endo et al. [70]
	XIAP protein and mRNA levels are higher in cancerous tissue compared to surrounding normal tissue.	Lee et al. [74], Guoan et al. [75]
cIAPs	cIAP1 is most frequently expressed in nucleus while cIAP2 in the cytoplasm. A role of nuclear localization is suggested to be involved in the pathogenesis of CRC.	Ponnelle et al. [71]
	Higher cIAP2 levels are observed in stage II CRC either lymphoid metastasis and higher rate of chemotherapy failure with FU.	Karasawa et al. [72]
	Together with TUCAN, cIAP2 low levels presented positive correlation with higher five-year survival.	Krajewska et al. [73]
Livin	Livin expression correlated with longer survival	Lee et al. [74]
Apollon	Apollon was observed overexpressed in CRC tissue and correlated with unfavorable clinical features.	Bianchini et al. [77]
Smac	Decreased expression of Smac was considered an independent factor for poor prognosis.	Endo et al. [78]
**Breast Carcinoma**		
XIAP	XIAP and survivin expression was observed to be increased in advanced cancer.	Pluta et al. [80]
	Increased in XIAP expression was observed in higher grades of ductal invasive breast carcinoma and ductal breast carcinoma in situ.	Yang et al. [81]
	High percentage of breast invasive ductal carcinoma with high immunoscore showed high XIAP expression.	Zhang et al. [82]
	Higher cytoplasmic expression is observed compared to normal tissues and correlated with other molecular abnormalities. It was considered a prognostic biomarker.	Xu et al. [83]
	High XIAP protein related with shorter OS	
cIAP2	Levels were higher in node positive breast cancer	Pluta et al. [80]
cIAP1	cIAP1 levels did not correlate with clinicopathological features	Pluta et al. [80]
Survivin	Correlated with metastasis, advanced stage and tumor size.	Youssef et al. [85]
	Positive expression of survivin have significant association with worse OS	Song et al. [88]
Livin	High expression is observed in high-invasive breast cancer cells compared to non-invasive cells. Authors described potential role in migration of cancer cells.	Li et al. [13]
Smac	Lower expression was observed compared to healthy tissue	Pluta et al. [89]
